# 1310. Provider and Facility Variation in Empiric Broad-Spectrum Antibiotic Use for Hospitalization Pneumonia: A Mixed Methods Study of Veterans Affairs Facilities

**DOI:** 10.1093/ofid/ofab466.1502

**Published:** 2021-12-04

**Authors:** Barbara E Jones, Peter Taber, Jian Ying, Jorie M Butler, McKenna Nevers, Makoto M Jones, Tom Greene, Vanessa W Stevens, Susan Zickmund, Charlene Weir, Matthew Samore

**Affiliations:** 1 University of Utah Health, Salt Lake City, UT; 2 University of Utah and VA Salt Lake City Healthcare System, Salt Lake City, Utah; 3 Division of Pulmonary & Critical Care Medicine, Department of Internal Medicine, University of Utah School of Medicine, Salt Lake City, Utah; 4 University of Utah, Salt Lake City, UT; 5 IDEAS Center of Innovation, VA Salt Lake City Health Care System, Salt Lake City, Utah

## Abstract

**Background:**

We previously found widespread variation in the empiric use of antibiotics against methicillin-resistant *Staph aureus* (anti-MRSA) and *Pseudomonas aeruginosa* (anti-PAER) for patients hospitalized for pneumonia. To explore this variation further, we conducted (1) quantitative analyses of facility-level versus provider-level variation, and (2) qualitative interviews with emergency department providers.

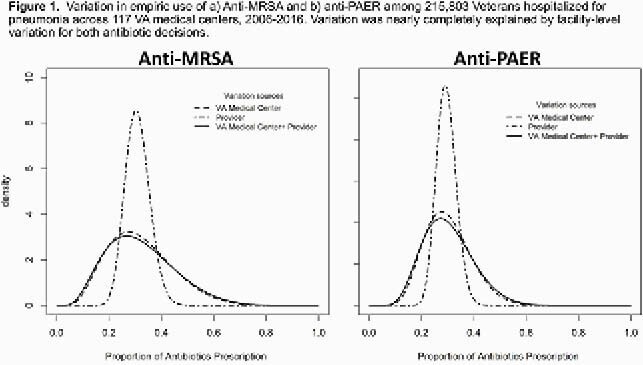

**Methods:**

For each hospitalization, we predicted the probability of anti-MRSA and anti-PAER use by fitting machine learning models from 75 patient variables. We estimated the predicted risk of anti-MRSA/anti-PAER and facility features among patients hospitalized at upper versus lower 10% facilities after controlling for patient characteristics. We plotted density curves with the variance attributed to facility and provider alone and together. We then interviewed 16 emergency department (ED) providers at 8 VA facilities using a cognitive task analysis.

**Results:**

Among 215,803 hospitalizations at 128 VA facilities 1/1/2006-12/31/2016, 31% received empiric anti-MRSA and 29% received empiric anti-PAER antibiotics. Hospitalizations at upper-decile facilities had a 50% and 45% adjusted probability of receiving anti-MRSA and anti-PAER antibiotics, compared to 15% and 20% in the lower-decile facilities. Facility features most predictive of anti-MRSA or anti-PAER use after adjusting for patient characteristics were complexity level (33% and 30% in high versus 15% and 20% in low complexity facilities). Variation in empiric anti-MRSA and anti-PAER use was almost completely at the facility level (**Figure 1**). Providers reported social influences from the opinions of other providers during decision-making and a high trust in guidelines and order sets. Consideration of pathogens was not mentioned by any providers at high-prescribing facilities.

**Conclusion:**

Variation in empiric use of anti-MRSA and anti-PAER antibiotics in pneumonia clustered nearly completely at the facility level. ED providers report social influences during decision-making and a high trust in guidelines and order sets. Guidelines, order sets, and facility-level clinical champions that promote consideration of pathogens could be important strategies for de-adoption.

**Disclosures:**

**All Authors**: No reported disclosures

